# HDAC Class I Inhibitor Domatinostat Induces Apoptosis Preferentially in Glioma Stem Cells Through p53-Dependent and -Independent Activation of BAX Expression

**DOI:** 10.3390/ijms26167803

**Published:** 2025-08-13

**Authors:** Yurika Nakagawa-Saito, Yasufumi Ito, Kazuki Nakamura, Yuta Mitobe, Keita Togashi, Shuhei Suzuki, Senri Takenouchi, Asuka Sugai, Yukihiko Sonoda, Chifumi Kitanaka, Masashi Okada

**Affiliations:** 1 Department of Molecular Cancer Science, Yamagata University School of Medicine, 2-2-2 Iida-nishi, Yamagata 990-9585, Japan; 2 Department of Obstetrics and Gynecology, Yamagata University School of Medicine, 2-2-2 Iida-nishi, Yamagata 990-9585, Japan; 3 Department of Neurosurgery, Yamagata University School of Medicine, 2-2-2 Iida-nishi, Yamagata 990-9585, Japan; 4 Department of Ophthalmology and Visual Sciences, Yamagata University School of Medicine, 2-2-2 Iida-nishi, Yamagata 990-9585, Japan; 5 Department of Clinical Oncology, Yamagata Prefectural Shinjo Hospital, 720-1 Kanazawa, Shinjo 996-8585, Japan; 6 Research Institute for Promotion of Medical Sciences, Yamagata University Faculty of Medicine, 2-2-2 Iida-nishi, Yamagata 990-9585, Japan

**Keywords:** glioma-initiating cells, brain tumor, HDACi, 4SC-202, Bcl-2-associated X protein

## Abstract

Domatinostat is an inhibitor of class I histone deacetylases, whose safety and efficacy as a cancer therapeutic has been demonstrated in a recent phase II study in patients with esophagogastric adenocarcinoma. We previously showed that domatinostat exhibited preferential cytotoxic activity against glioma stem cells (GSCs) compared to their differentiated counterparts. However, the underlying mechanism behind the preferential cytotoxicity is yet to be elucidated. In this study, we examined the effects of domatinostat treatment, as well as those of the knockdown of p53 or BAX or of the overexpression of BAX, on the expression of p53, BAX, and cleaved caspase substrates and on cell death in GSCs and their isogenic, differentiated counterparts. The results obtained indicated that domatinostat induced caspase-dependent apoptotic cell death preferentially in GSCs, which was accompanied by increased BAX expression in GSCs, but not in their differentiated counterparts. The increased BAX expression was required for domatinostat-induced GSC death, whereas BAX overexpression was sufficient to induce cell death in both GSCs and their differentiated counterparts. Notably, the expression of BAX after domatinostat treatment showed an early, p53-independent increase followed by a late, p53-dependent one. Together, the results suggest that the unique ability of domatinostat to activate the p53-dependent and -independent programs of BAX expression selectively in GSCs could account for its preferential cytotoxicity against GSCs. Our findings may also help guide the selection of patients with glioblastoma, and possibly those with other types of cancer, who are most likely to benefit from domatinostat treatment and optimize the treatment strategy for such patients.

## 1. Introduction

Glioblastoma is the most malignant type of glioma and one of the most aggressive cancers among all human malignancies. The standard treatment for glioblastoma consists of surgical resection followed by chemoradiotherapy, but even with optimal treatment, recurrence of the disease is nearly inevitable [1]. Consequently, the 5-year survival rate remains as low as 7.1%, according to recent statistics [2].

Glioma stem cells (GSCs), which are cancer stem cells in glioblastoma, are a small subpopulation that are characterized by stronger treatment resistance and a higher self-renewal capacity than non-GSCs and also contribute to tumor recurrence and resistance to treatment. Therefore, the targeting of GSCs is a promising approach to prevent recurrence and improve the long-term prognosis of glioblastoma [3,4,5,6]. Recent studies revealed that GSCs possess unique vulnerabilities that are not shared by their differentiated, non-GSC counterparts, suggesting the potential for therapeutic strategies based on GSC-specific vulnerabilities [7,8,9].

Histone deacetylases (HDACs) reduce the acetylation of histones and induce gene repression, thereby leading to cancer [10]. Previous studies reported that HDACs were abundantly expressed in GSCs, in support of the possible role of HDACs in the pathogenesis of glioblastoma [11,12,13]. Thus, HDACs may possibly be potential candidates for GSC-specific vulnerabilities, which can then be targeted with HDAC inhibitors, drugs that promote the acetylation of histones and open chromatin conformation at tumor suppressor gene loci and contribute to tumor suppression [10]. However, HDAC inhibitors have had limited clinical success in the treatment of glioblastoma due to their high toxicity and low specificity, with valproic acid being the only HDAC inhibitor that has advanced to a phase III clinical trial (NCT03243461) [14].

We previously demonstrated that domatinostat (4SC-202), a specific inhibitor of class I HDACs 1–3, not only preferentially inhibited the proliferation and survival of GSCs, but also reduced their stemness, suggesting that class-specific HDAC inhibitors could successfully target the unique vulnerabilities of GSCs [15]. In support of the therapeutic efficacy and safety of domatinostat, a recent phase II clinical trial on esophagogastric adenocarcinoma and colorectal cancer showed that domatinostat did not exhibit dose-limiting toxicity (Cmax ~490 nM), and promising findings were obtained for esophagogastric adenocarcinoma (NCT03812796) [16]. However, the molecular mechanism behind the GSC-specific action of domatinostat still remains elusive. In the present study, using in vitro models of GSCs and their differentiated counterparts, we addressed this question and revealed that domatinostat induces BAX expression through both p53-dependent and -independent pathways, preferentially in GSCs, highlighting a novel mechanism of action of domatinostat against GSCs, which are considered one of the root causes of poor prognosis in glioblastoma.

## 2. Results

### 2.1. Differential Pro-Apoptotic Effects of Domatinostat on GSCs and Their Differentiated Counterparts

**Figure 1:**
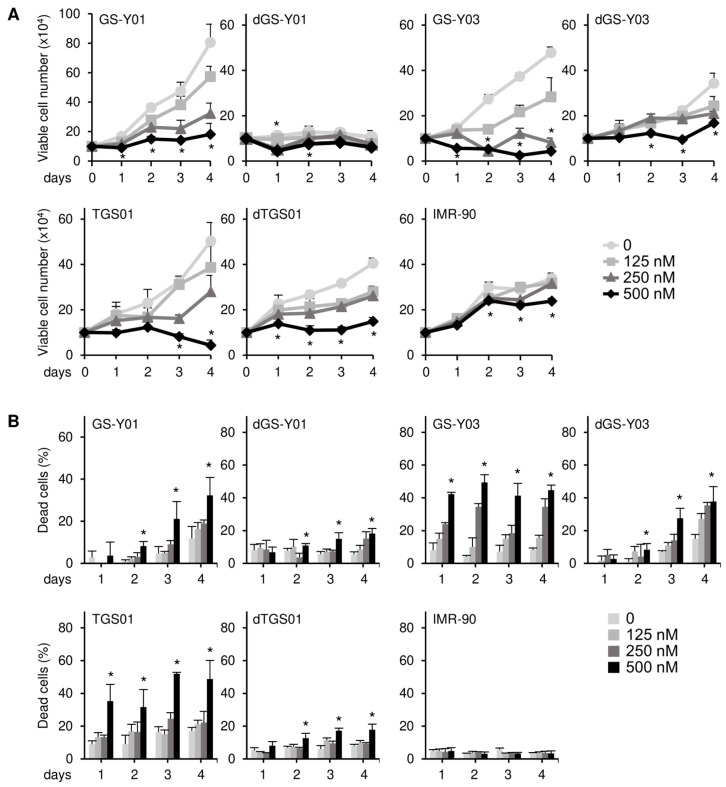
Domatinostat preferentially inhibits cell growth and induces cell death in glioma stem cells. (**A**) GS-Y01, GS-Y03, TGS01, their differentiated counterparts (dGS-Y01, dGS-Y03, and dTGS01), and IMR-90 human lung fibroblasts were treated with the indicated concentrations of domatinostat for 1–4 days, and the number of viable cells was assessed by trypan blue dye exclusion. (**B**) Cells were treated as in (**A**), and the percentage of dead cells was evaluated using the trypan blue dye exclusion assay. * *p* < 0.05 vs. treatment without domatinostat (i.e., at 0 nM).

**Figure 2:**
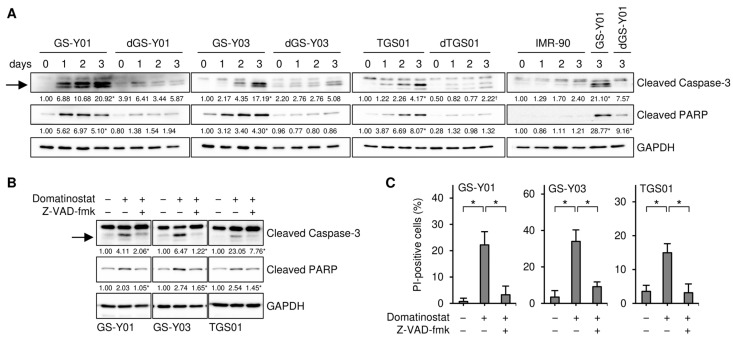
Domatinostat induces apoptotic cell death. (**A**) GS-Y01, GS-Y03, TGS01, their differentiated counterparts (dGS-Y01, dGS-Y03, and dTGS01), and IMR-90 human lung fibroblasts were treated without or with 500 nM domatinostat for 1–3 days, and were then subjected to Western blot analyses of the indicated proteins. The numbers below the images represent the means (*n* = 2) of the relative band intensities after each band was quantified by densitometry and normalized to the GAPDH value. * *p* < 0.05 vs. cells before domatinostat treatment (i.e., cells at 0 days) for each of the glioma stem cell lines and their differentiated counterparts, except for the right most panel where comparisons were made with the adjacent left lane (i.e., IMR-90 treated with domatinostat for 3 days). (**B**) Cells were treated without or with 500 nM domatinostat in the absence or presence of 40 μM Z-VAD-fmk for 1 day, and were then subjected to Western blot analyses of the indicated proteins. * *p* < 0.05 vs. cells treated with domatinostat alone. (**C**) Cells were treated as in (**B**), and were then subjected to the propidium iodide (PI) incorporation assay to assess the percentage of dead cells. * *p* < 0.05. In (**A**,**B**), the expected migrating position for cleaved caspase-3 is indicated by an arrow.

We previously reported through a concentration-dependent analysis that domatinostat inhibited the growth of GSCs more efficiently than that of their differentiated counterparts [15]. We aimed to confirm these findings by conducting a time-course analysis in the present study. Consistent with the previous study [15], the results of the time-course analysis revealed the concentration-dependent growth inhibition of GSCs by domatinostat at each time point examined, which was less pronounced in their differentiated counterparts (Figure 1A). Similarly, domatinostat induced cell death in GSCs in a concentration-dependent manner at each time point examined, which was more efficient than that in their differentiated counterparts (Figure 1B), but did not affect IMR-90 normal human fibroblasts. Our previous findings showed that domatinostat-induced GSC death was accompanied by the activation of the caspase pathway; however, it remains unknown whether this cell death requires caspase activation for its execution [15]. The results of the time-course analysis of cleaved caspase-3 and PARP expression indicated that the caspase pathway was efficiently activated by domatinostat in GSCs, less efficiently in their differentiated counterparts, and was not activated in IMR-90 fibroblasts (Figure 2A). The pan-caspase inhibitor Z-VAD-fmk inhibited the domatinostat-induced cleavage of caspase-3 and PARP (Figure 2B), and nearly abolished domatinostat-induced GSC death (Figure 2C). Collectively, these results suggest that domatinostat induced caspase-dependent apoptosis preferentially in GSCs over their differentiated counterparts.

### 2.2. BAX Expression Selectively Increases in GSCs After the Domatinostat Treatment and Is Required for the Domatinostat-Induced Apoptotic Death of GSCs

**Figure 3:**
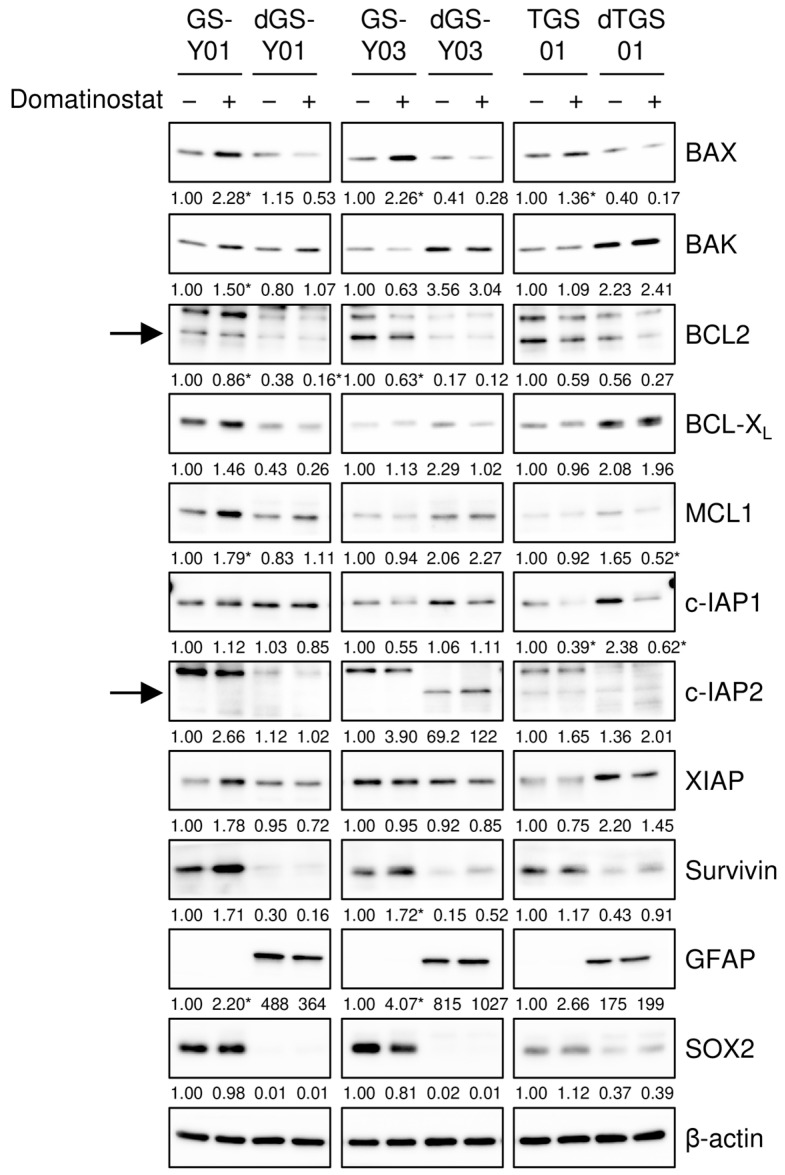
Domatinostat increases BAX expression selectively in glioma stem cells. The indicated glioma stem cells and their differentiated counterparts were treated without or with 500 nM domatinostat for 1 day, and were then subjected to Western blot analyses of the indicated proteins. The expected migrating positions for BCL2 and c-IAP2 are indicated by arrows. The numbers below the images represent the means (*n* = 2) of the relative band intensities after each band was quantified by densitometry and normalized to the β-actin value. * *p* < 0.05 vs. cells treated without domatinostat for each of the glioma stem cell lines and their differentiated counterparts.

**Figure 4:**
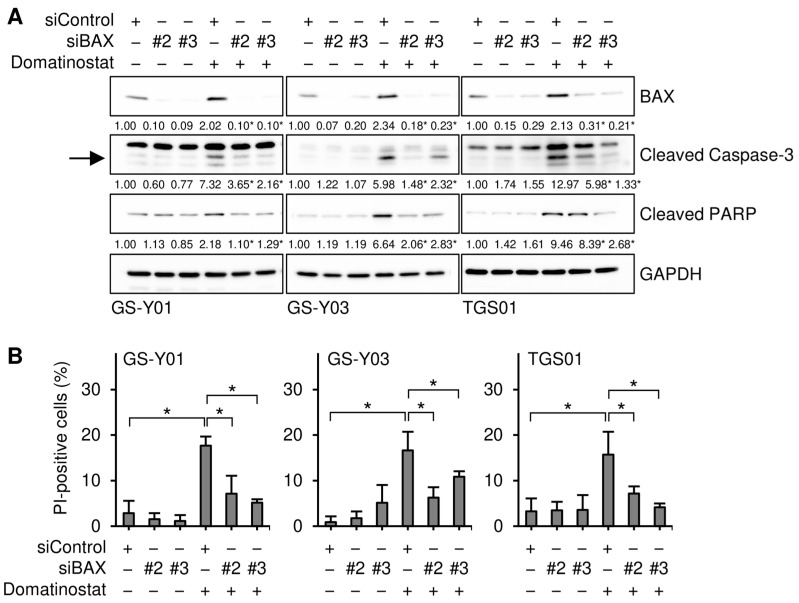
BAX is required for the domatinostat-induced apoptotic death of glioma stem cells. (**A**) Cells were transiently transfected with either an siRNA against BAX (siBAX) or a control RNA (siControl). One day after transfection, cells were treated without or with 500 nM domatinostat for 1 day, and were then subjected to Western blot analyses of the indicated proteins. The expected migrating position for cleaved caspase-3 is indicated by an arrow. The numbers below the images represent the means (*n* = 2) of the relative band intensities after each band was quantified by densitometry and normalized to the GAPDH value. * *p* < 0.05 vs. cells transfected with siControl and treated with domatinostat. (**B**) Cells were transfected and treated as in (**A**), and were then subjected to the PI incorporation assay to assess the percentage of dead cells. * *p* < 0.05.

To gain insights into the molecular mechanisms underlying the selective pro-apoptotic effects of domatinostat on GSCs, we monitored the expression of core components of the apoptotic machinery in GSCs and their differentiated counterparts in the presence and absence of the domatinostat treatment. We found that the expression of BAX, a pro-apoptotic member of the BCL2 protein family, was increased by the domatinostat treatment in all the GSC lines examined, whereas BAX expression remained unchanged in the differentiated counterparts (Figure 3). To investigate the possible involvement of BAX in the domatinostat-induced apoptotic death of GSCs, we examined the effects of BAX knockdown on the caspase pathway and cell death in GSCs. The introduction of siRNAs directed against non-overlapping regions of the human BAX gene into GSCs effectively inhibited caspase activation (Figure 4A), as well as cell death (Figure 4B) induced by domatinostat.

### 2.3. An Increase in BAX Expression Is Required for Domatinostat to Activate the Apoptotic Caspase Pathway

**Figure 5:**
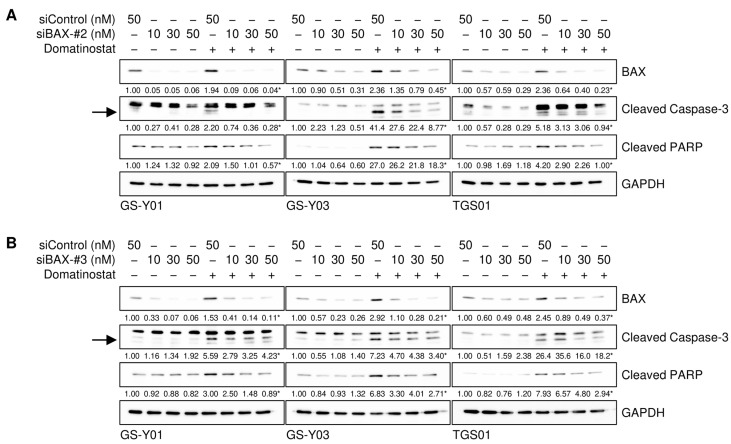
The incremental inhibition of BAX attenuates domatinostat-induced caspase activation. (**A**,**B**) Cells were transiently transfected with a control RNA (siControl) or an siRNA against BAX (siBAX-#2 in (**A**) and siBAX-#3 in (**B**)) at the indicated concentrations. One day after transfection, cells were treated without or with 500 nM domatinostat for one day, and were then subjected to Western blot analyses of the indicated proteins. The expected migrating positions for cleaved caspase-3 are indicated by arrows. The numbers below the images represent the means (*n* = 2) of the relative band intensities after each band was quantified by densitometry and normalized to the GAPDH value. * *p* < 0.05 vs. cells transfected with siControl and treated with domatinostat.

The results presented so far suggest that domatinostat induced the apoptotic death of GSCs by increasing the expression of BAX. However, it remains unclear whether BAX contributes to apoptotic caspase activation in GSCs in a quantitative (i.e., through increased expression) or qualitative (i.e., a certain level of expression required) manner. To distinguish between these possibilities, we knocked down BAX incrementally by modulating the concentration of siRNAs introduced into GSCs and assessed the impact of gradual changes in BAX expression levels on the domatinostat-induced activation of the caspase pathway. The results obtained showed that cleaved caspase-3 and PARP levels paralleled those of BAX expression, suggesting that the caspase pathway was activated in a BAX expression level-dependent manner (Figure 5A,B).

### 2.4. Increased BAX Expression in the Absence of Domatinostat Is Sufficient to Activate the Apoptotic Caspase Pathway in GSCs

**Figure 6:**
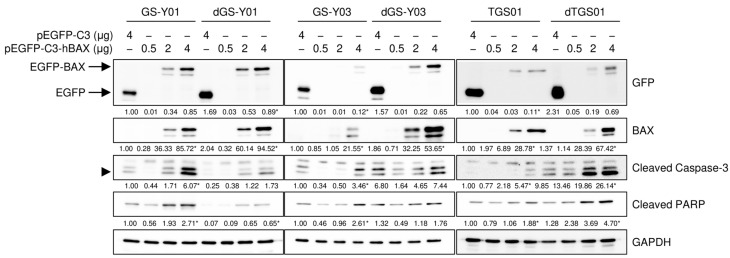
The forced overexpression of BAX is sufficient to induce apoptotic caspase activation. Cells were transiently transfected with a plasmid expressing human BAX (pEGFP-C3-hBAX) or the control plasmid (pEGFP-C3) at the indicated amounts. One day after transfection, cells were subjected to Western blot analyses of the indicated proteins. The expected migrating position for cleaved caspase-3 is indicated by an arrowhead. The numbers below the images represent the means (*n* = 2) of the relative band intensities after each band was quantified by densitometry and normalized to the GAPDH value. * *p* < 0.05 vs. cells transfected with pEGFP-C3 for each of the glioma stem cell lines and their differentiated counterparts.

We then investigated whether BAX activated the caspase pathway in GSCs, even in the absence of the domatinostat treatment. To achieve this, we introduced varying amounts of the expression vector that drives the expression of BAX fused to GFP and achieved different levels of BAX expression in GSCs. The expression levels of cleaved caspase-3 and PARP increased upon BAX expression in the absence of domatinostat and were parallel to those of BAX expression, demonstrating that BAX alone activated the caspase pathway in an expression level-dependent manner (Figure 6). Furthermore, BAX overexpression activated the caspase pathway in a similar manner in differentiated GSCs (Figure 6). Collectively, these results suggest that the differential sensitivity of GSCs and their differentiated counterparts to domatinostat reflected the ability and inability of the former and latter, respectively, to express BAX in response to the domatinostat treatment and not their differential sensitivity to the BAX-mediated activation of the apoptotic program.

### 2.5. Domatinostat Induces BAX Expression in Both p53-Dependent and -Independent Manners in GSCs

**Figure 7:**
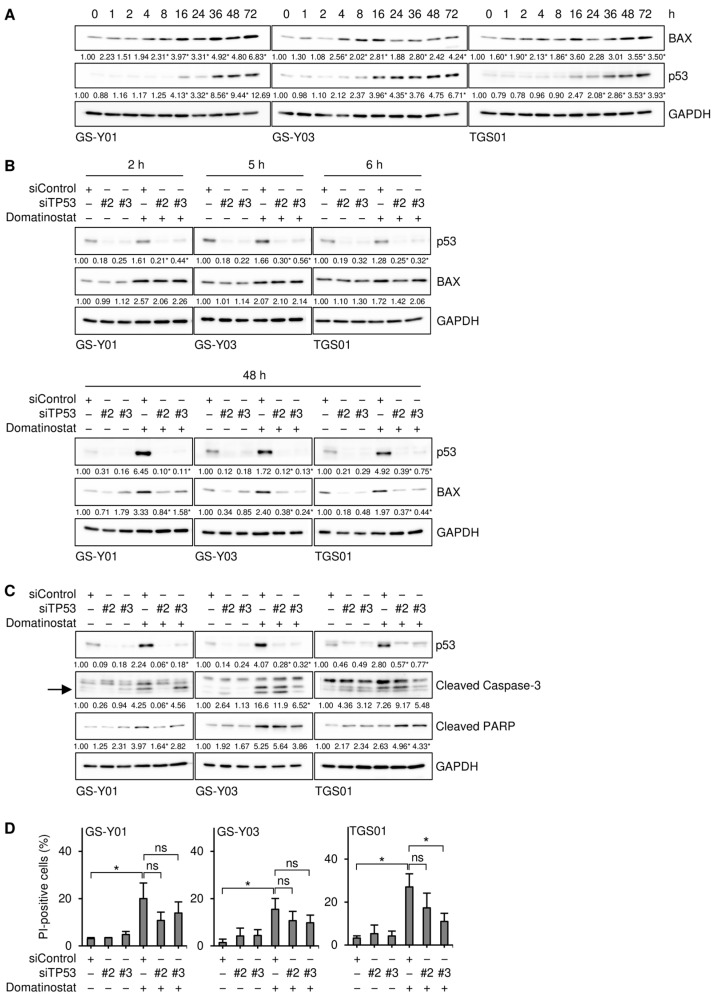
Domatinostat induces the expression of BAX in both p53-dependent and -independent manners in glioma stem cells. (**A**) Cells were treated without or with 500 nM domatinostat for the indicated hours, and were then subjected to Western blot analyses of the indicated proteins. The numbers below the images represent the means (*n* = 2 for (**A**), *n* = 3 for the others) of the relative band intensities after each band was quantified by densitometry and normalized to the GAPDH value. * *p* < 0.05 vs. cells treated without domatinostat (i.e., at 0 h). (**B**,**C**) Cells were transiently transfected with an siRNA against TP53 (siTP53) or a control RNA (siControl). One day after transfection, cells were treated without or with 500 nM domatinostat for the indicated hours (**B**) or for 1 day (**C**), and were then subjected to Western blot analyses of the indicated proteins. The expected migrating position for cleaved caspase-3 is indicated by an arrow. * *p* < 0.05 vs. cells transfected with siControl and treated with domatinostat. (**D**) Cells were transfected and treated as in (**C**), and were then subjected to the PI incorporation assay to assess the percentage of dead cells. * *p* < 0.05.

We examined the mechanisms by which domatinostat induced BAX expression in GSCs. Our preliminary results showed that domatinostat induced the expression of p53 in GSCs. Since the BAX gene is a transcriptional target of p53, we investigated whether p53 mediated BAX expression induced by domatinostat in GSCs. We conducted a time-course analysis of p53 and BAX expression to establish whether their expression patterns correlated with each other. The results obtained revealed that in all of the GSC lines examined, the expression of BAX exhibited a multi-phasic pattern with two peaks at early and late time points, whereas that of p53 increased monotonically within the observation period (~72 h after the domatinostat treatment). Importantly, the earlier peak in BAX expression after the domatinostat treatment was observed well before the increase in p53 expression, suggesting its independence of p53 (Figure 7A). Therefore, we investigated whether BAX expression at the earlier and later time points after the domatinostat treatment was dependent on p53. The results of knockdown experiments showed that upon the knockdown of p53, BAX expression remained unchanged at the early time point and decreased at the late time point, suggesting that the dependency of domatinostat-induced BAX expression on p53 varied with time after the domatinostat treatment (Figure 7B). We also examined the role of p53 in the domatinostat-induced apoptotic death of GSCs. The knockdown of p53 resulted in slight decreases in caspase activation and cell death (Figure 7C,D), which may reflect the essential and non-essential roles of p53 in domatinostat-induced BAX expression.

## 3. Discussion

In our previous study, we demonstrated for the first time that domatinostat, a selective class I HDAC inhibitor with established human safety data from clinical trials, preferentially induced cell death in GSCs [15]; however, the underlying mechanisms were unclear. In the present study, by directly comparing GSCs with their differentiated counterparts, we demonstrated that domatinostat selectively induced BAX expression-dependent apoptotic death in GSCs. Notably, the forced expression of BAX not only activated the caspase pathway in GSCs, but also in their differentiated counterparts, suggesting that the differential ability of domatinostat to induce BAX expression between GSCs and their differentiated counterparts accounts for the selective effect of domatinostat.

Previous studies have shown that p53 plays a key role in the transcriptional regulation of the BAX gene [17]. In the present study, time-course analyses of BAX and p53 expression in GSCs treated with domatinostat revealed a biphasic pattern of BAX up-regulation, comprising an early phase preceding the increase in p53 expression and a late phase coinciding with it. Furthermore, p53 knockdown experiments demonstrated that only the late-phase increase in BAX expression was dependent on p53. However, the molecular mechanisms underlying the early-phase p53-independent up-regulation of BAX induced by domatinostat remains unclear.

One possible explanation is that domatinostat induces changes in histone acetylation at the BAX gene promoter, thereby affecting its transcription in a p53-independent manner and changing BAX expression levels [18,19,20]. In this context, it is important to note that HDAC inhibition was shown to induce early changes in H4 acetylation near the BAX gene promoter (within 12 h), facilitating transcription factor access and promoting BAX up-regulation, even in p53-null tumor cells [19]. In addition, our preliminary data show that the treatment of GSCs with domatinostat induces a global increase in histone H4 acetylation at approximately the same time as the early-phase up-regulation of BAX (unpublished observations). Therefore, histone acetylation may have been induced early after the domatinostat treatment, resulting in more accessible BAX gene promoter and thereby allowing transcriptional activation by transcription factors other than p53, such as Sp1.

In support of the role of Sp1 in this scenario, HDAC inhibitors have been shown to promote the recruitment of endogenous Sp1 to promoter regions, thereby enhancing gene expression [21,22]. Furthermore, our preliminary data also show that Sp1 is more highly expressed in GSCs than in their differentiated counterparts (unpublished observations). Collectively, these findings together with the present results suggest that the early up-regulation of BAX expression specifically observed in GSCs treated with domatinostat may be attributable to increased histone acetylation at the BAX gene promoter, leading to transcriptional activation by Sp1, which is highly expressed in GSCs. Future studies are needed to investigate this potential mechanism in more detail.

On the other hand, the increase in p53 following the domatinostat treatment was more pronounced in GSCs than in their differentiated counterparts. Moreover, we detected elevated p53 protein levels without a corresponding increase in p53 mRNA levels (unpublished observations), suggesting the presence of a GSC-specific mechanism for p53 protein stabilization induced by domatinostat. We previously reported that the expression of MDM2, a p53-degrading enzyme, was higher in GSCs than in their differentiated counterparts [23]. In addition, the interaction between MDM2 and HDAC1 was found to enhance the deacetylation of p53, thereby reducing its protein stability [24]. Conversely, the inhibition of HDAC1 in GSCs has been reported to increase p53 acetylation and stabilize the p53 protein [11]. These findings indicate that the GSC-selective, p53-dependent late-phase up-regulation of BAX expression induced by domatinostat may be mediated by interactions between the highly expressed MDM2-HDAC1 complex and p53 in GSCs. This potential mechanism also warrants further investigation in future studies.

Epigenetic dysregulation frequently occurs in cancer cells, including glioblastoma, and HDAC inhibitors have attracted attention as promising therapeutic agents capable of reactivating tumor suppressor factors that have been inactivated due to these abnormalities [25,26]. However, among clinical trials targeting glioblastoma, the only HDAC inhibitor that has progressed to a phase III trial is valproic acid (NCT03243461), and their high toxicity and low selectivity are considered to be major reasons for the limited success of other compounds [14]. In contrast, domatinostat is a selective inhibitor of HDACs 1–3 that is orally available [27], and in a phase II clinical trial targeting esophagogastric and colorectal adenocarcinomas, no dose-limiting toxicities were reported [16]. Notably, favorable outcomes were obtained, particularly for esophagogastric adenocarcinoma (NCT03812796), suggesting that domatinostat is currently one of the most promising HDAC inhibitors for clinical application [16]. In considering clinical application of domatinostat for the treatment of glioblastoma, it is also of interest whether domatinostat can cross the blood–brain barrier (BBB), at which efflux transporters excrete anticancer agents and tight junctions between the capillary endothelial cells block their entry [28]. Currently, there are no data available on the BBB penetrability of domatinostat. However, since the BBB is known to be disrupted in brain tumors [29,30], domatinostat is expected to at least cross the BBB and reach GSCs present in the tumor bulk. Future preclinical animal studies are therefore warranted to investigate the therapeutic effect, as well as the mechanism, of domatinostat in orthotopic models of glioblastoma.

In the present study, we found that domatinostat induced cell death in GSCs via both the p53-dependent and -independent up-regulation of BAX expression. Therefore, the pharmacological effects of domatinostat appear to be affected by the status of the BAX and TP53 genes in the targeted cells.

A previous study reported that the BAX gene was frequently mutated in cancers harboring defects in DNA mismatch repair (dMMR), and that these mutations often involved frameshift mutations in a tract of eight deoxyguanosines [(G)8] within the BAX gene [31,32]. However, the frequency of microsatellite instability-high (MSI-high), which results from dMMR, was low in glioblastoma [33,34], and even in gliomas with a high frequency of MSI-high, BAX gene mutations were rare [35]. In addition to frameshift mutations caused by dMMR, mutations in the promoter region of the BAX gene have been reported to contribute to reduced BAX expression in chronic lymphocytic leukemia, head and neck squamous cell carcinoma, and breast cancer [36,37,38]. However, to the best of our knowledge, there has been no such report for glioblastoma. Therefore, in glioblastoma, inactivating mutations in the BAX gene are considered to be extremely rare [39], suggesting that domatinostat is a highly promising therapeutic candidate for this tumor type.

Furthermore, TP53 mutations are present in fewer than 30% of glioblastoma cases, while the remaining majority of cases retain wild-type TP53 [40,41,42]. As discussed above, domatinostat-induced BAX-dependent cell death occurs via both p53-dependent and -independent mechanisms. Therefore, domatinostat is expected to exert therapeutic effects not only in GSCs harboring wild-type TP53, but also in those with TP53 mutations.

In summary, this is the first study to investigate the mechanisms by which domatinostat preferentially induces cell death in GSCs. We found that domatinostat selectively increased BAX expression in GSCs through both p53-dependent and -independent mechanisms, thereby inducing GSC-specific cell death. These results may help guide the selection of optimal patient populations for clinical applications and also suggest that the therapeutic efficacy could be enhanced by combining domatinostat with other existing drugs that target non-GSC populations. Domatinostat, which targets the GSCs implicated in glioblastoma recurrence and treatment resistance, holds promise as a candidate therapeutic agent for achieving long-term survival in patients with glioblastoma.

## 4. Materials and Methods

### 4.1. Reagents and Antibodies

Domatinostat (4SC-202; S7555) was purchased from Selleck Chemicals (Houston, TX, USA). Z-VAD-fmk (3188-v) was purchased from Peptide Institute, Inc. (Osaka, Japan). Antibodies against Bax (#5023), Bak (#12105), c-IAP1 (#7065), c-IAP2 (#3130), cleaved caspase-3 (#9664), cleaved PARP (#9541), GAPDH (#5174), GFAP (#3670), survivin (#2808), and XIAP (#2045) were purchased from Cell Signaling Technology, Inc. (Beverly, MA, USA). Antibodies against Bcl-2 (sc-7382), GFP (sc-9996), Mcl-1 (sc-20679), and p53 (sc-126) were purchased from Santa Cruz Biotechnology, Inc. (Dallas, TX, USA). An antibody against SOX2 (MAB2018) was purchased from R&D Systems, Inc. (Minneapolis, MN, USA). An antibody against Bcl-XL (10783-1-AP) was purchased from ProteinTech Group, Inc. (Rosemont, IL, USA). An antibody against β-actin (A1978) was purchased from Merck KGaA (Darmstadt, Germany).

### 4.2. Cell Culture

The isolation and establishment of patient-derived GSCs (GS-Y01, GS-Y03, and TGS01) were conducted as previously described [43,44,45]. GSCs were maintained under previously reported monolayer stem cell culture conditions [46,47]. The differentiation of GSCs was induced by culturing cells in DMEM/F-12 medium supplemented with 10% fetal bovine serum (FBS, Thermo Fisher Scientific Inc., Waltham, MA, USA), 100 units/mL of penicillin, and 100 μg/mL of streptomycin for 7–14 days [47]. IMR-90, a human normal fetal lung fibroblast cell line, was purchased from the American Type Culture Collection (Manassas, VA, USA) and maintained in DMEM supplemented with 10% FBS. All IMR-90 experiments were performed using cells with a low passage number (<8).

### 4.3. Trypan Blue Dye Exclusion Assay

The numbers of viable and dead cells were assessed using the trypan blue dye exclusion assay [15]. Cells treated with domatinostat as described were pipetted (GSCs) or trypsinized (differentiated GSCs and IMR-90) and suspended in phosphate-buffered saline (PBS), and cells were then stained with 0.2% trypan blue solution (Merck) for 1 min. Viable and dead cells were identified by their ability and inability, respectively, to exclude trypan blue using a hemocytometer. The assay was repeated at least twice with similar results, and one set of representative data is presented.

### 4.4. PI Incorporation Assay

The PI incorporation assay was performed to assess the percentage of dead cells as previously described [15]. Cells treated with domatinostat as indicated in the figure legends were incubated with PI (1 mg/mL, Thermo Fisher Scientific) and Hoechst33342 (10 mg/mL, Thermo Fisher Scientific) at 37 °C for 5 min. To calculate the ratio of PI-positive cells (dead cells) to Hoechst-positive cells (total cells), fluorescent images were obtained using a fluorescence microscope (CKX53; EVIDENT, Tokyo, Japan) equipped with an iPhone 7 (Apple Inc., Cupertino, CA, USA) and scored. More than three areas of cells were counted to calculate the percentage of PI-positive cells. The assay was repeated at least twice with similar results, and one set of representative data is presented.

### 4.5. Western Blot Analysis

A Western blot analysis was conducted as previously described [8]. Cells were collected and washed with ice-cold PBS and lysed in RIPA buffer (10 mM Tris-HCl (pH 7.4), 0.1% sodium dodecyl sulfate (SDS), 0.1% sodium deoxycholate, 1% Nonidet P-40, 150 mM NaCl, 10 mM sodium fluoride, 10 mM sodium pyrophosphate, 1.5 mM sodium orthovanadate, 1 mM EDTA, and protease inhibitor cocktail set III (FUJIFILM Wako Pure Chemical Corporation, Osaka, Japan)). Lysates were immediately mixed with the same volume of 2 × Laemmli buffer (125 mM Tris-HCl (pH 6.8), 4% SDS, and 10% glycerol) and boiled at 95 °C for 10 min. The protein concentrations of the cell lysates were measured using a BCA protein assay kit (Thermo Fisher Scientific). Samples containing equal amounts of protein were separated by SDS-polyacrylamide gel electrophoresis and transferred to polyvinylidene difluoride membranes. The membranes were probed with the indicated primary antibodies followed by appropriate horseradish peroxidase (HRP)-conjugated secondary antibodies (Jackson ImmunoResearch Inc., West Grove, PA, USA), as recommended by the manufacturer of each antibody. To reprobe immunoblots, antibodies were stripped from the probed membranes using stripping buffer (2% SDS, 100 mM β-mercaptoethanol, and 62.5 mM Tris-HCl (pH 6.8)). After stripping, the membranes were washed with Tris-buffered saline with Tween 20, blocked with skim milk, and reprobed with appropriate antibodies. Immunoreactive bands were visualized using Immobilon Western Chemiluminescent HRP Substrate (Merck) and detected by a ChemiDoc Touch device (Bio-Rad Laboratories, Inc., Hercules, CA, USA). The analysis was repeated at least twice with similar results, and one set of representative data is presented.

### 4.6. Transfection of siRNAs and Plasmids

siRNAs against BAX (#2: HSS141355 and #3: HSS141356, Thermo Fisher Scientific) and p53 (TP53, #2: HSS186390 and #3: HSS186391, Thermo Fisher Scientific) or AllStars Negative Control siRNA (QIAGEN, Venlo, The Netherlands) were transfected using Lipofectamine RNAiMAX (Thermo Fisher Scientific) in accordance with the manufacturer’s instructions. The plasmid hBax C3-EGFP, a gift from Richard Youle (pEGFP-C3-hBAX: Addgene plasmid #19741; https://www.addgene.org/19741/ (accessed on 11 August 2025); RRID:Addgene_19741; Addgene, Watertown, MA, USA) [48], and the control plasmid pEGFP-C3 (GenBank accession #U57607; Takara Bio Inc., Shiga, Japan) were transiently transfected using Lipofectamine 2000 (Thermo Fisher Scientific) in accordance with the manufacturer’s instructions. Similar results were obtained from two independent biological replicates, and one set of representative data is presented.

### 4.7. Statistical Analysis

Data analyses were performed using the software Microsoft Excel 2019 (Version 2505, Redmond, WA, USA), and results are expressed as the mean value + standard deviation from at least triplicate samples. Additionally, the significance of differences was assessed using Student’s two-tailed *t*-test for comparisons of two groups. *p* values < 0.05 were considered to be significant and are indicated with asterisks in the figures.

## Data Availability

All data are contained in this article and there are no repository data.
